# Supplementary figures and images for: Transforaminal pulsed radiofrequency and epidural steroid injection on chronic lumbar radiculopathy: A prospective observational study from a tertiary care hospital in Vietnam
Source: PLoS One. 2024 Apr 4;19(4):e0292042. doi: 10.1371/journal.pone.0292042 (PMC10994338; doi:10.1371/journal.pone.0292042)

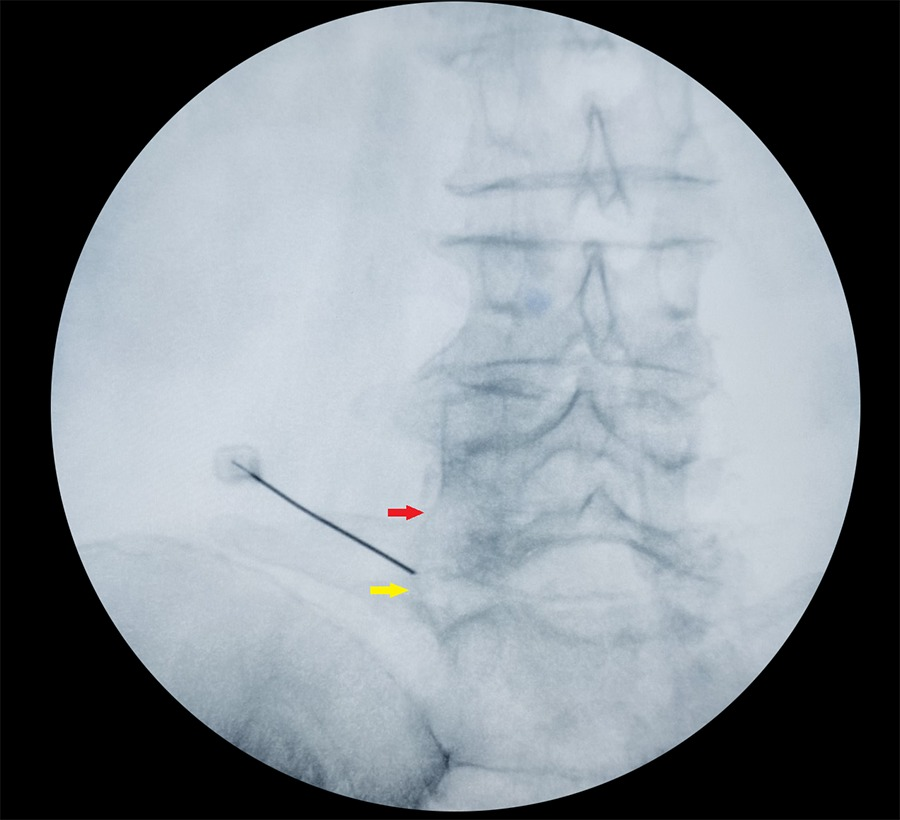

Supplement: S1 Fig — Red arrow: Hypertrophy of L4L5 facet join. RF canula was below the facet joint to enter inter-peduncle space. Yellow arrow: Inter-vertebrae foramen of L5S1 segment. (TIF) [file pone.0292042.s001.tif]

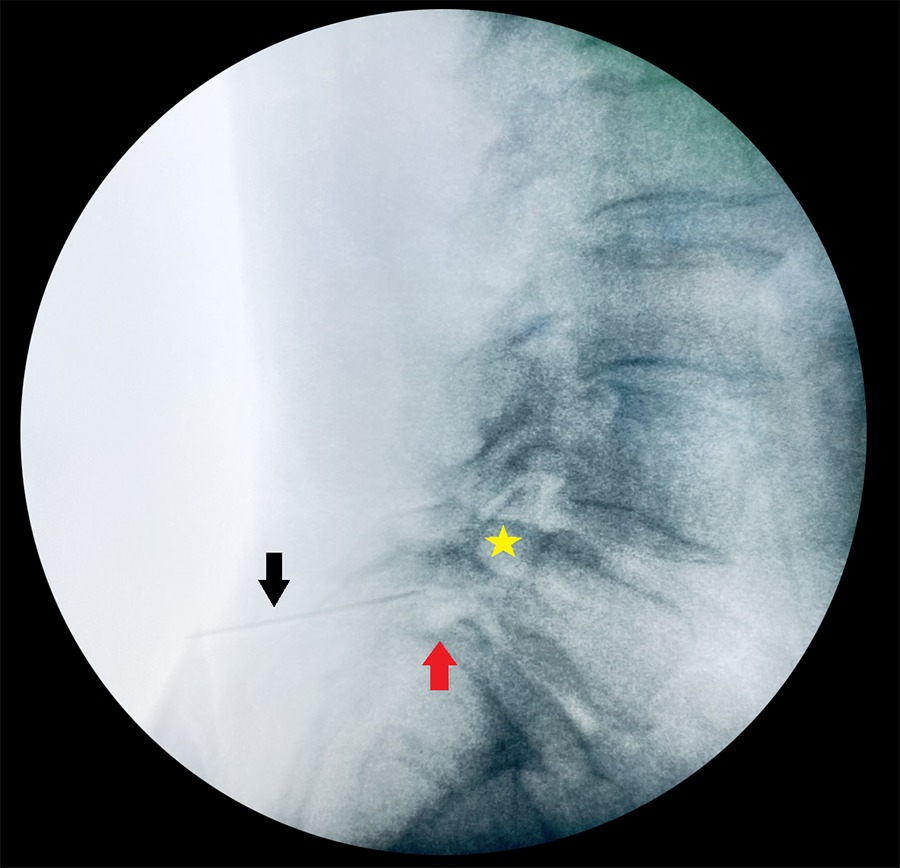

Supplement: S2 Fig — Red arrow: Inter-vertebrae foramen of L5S1 segment. Yellow star: Pedicle of L5 vertebrae. Black arrow: RF canula tip was check under C-arm. (TIF) [file pone.0292042.s002.tif]

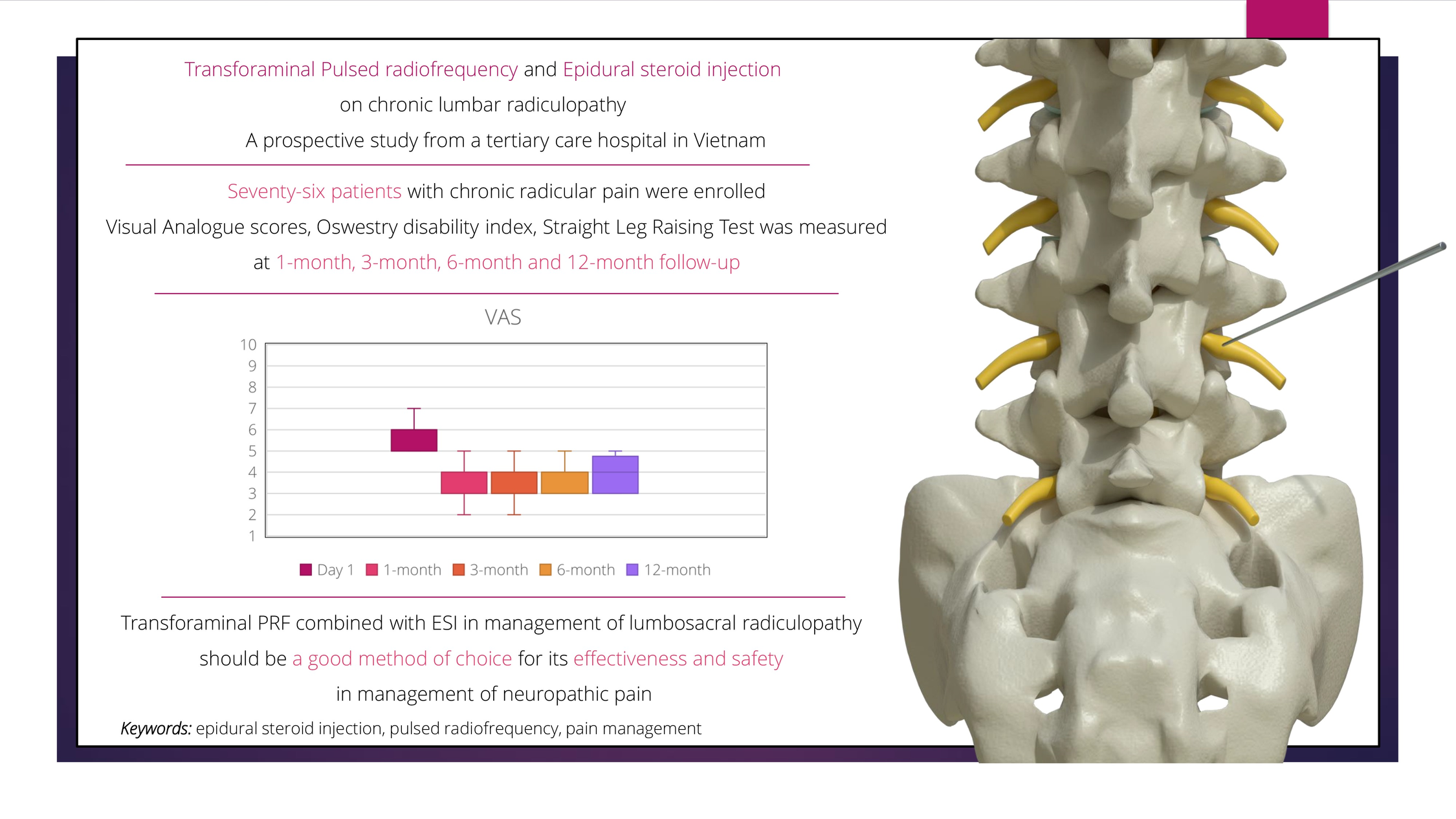

Supplement: S1 Graphical abstract — (TIF) [file pone.0292042.s003.tif]
