# Supplementary material for: Transforaminal pulsed radiofrequency and epidural steroid injection on chronic lumbar radiculopathy: A prospective observational study from a tertiary care hospital in Vietnam
Source: PLoS One. 2024 Apr 4;19(4):e0292042. doi: 10.1371/journal.pone.0292042 (PMC10994338; doi:10.1371/journal.pone.0292042)
Supplement: Attachment — Submitted filename: Response to reviewers comments.docx [file pone.0292042.s006.docx]

**Thank you for providing thoughtful and valuable feedback on the manuscript. We have responded to all reviewer’ comments below and have changed the text accordingly. We believe this has strengthened the manuscript.**

**REVIEWER 1**

*1. It is written as "neuropathic pain" in the abstract (conclusion) part. However, there is no neuropathic pain assessment scale in the material method section. For this reason, it would be more appropriate to say "pain" instead of neuropathic pain.*

**Response:** Thank you for your valuable comments. We have adjusted the term:

“Transforaminal PRF combined with ESI in management of lumbosacral radiculopathy should be a good method of choice for its effectiveness and safety in management of pain.” (page 2, line: 19-21).

*2. The phrase "A case series" in the title partially contradicts the statement "A prospective observational study" in the material method section. The title or abstract should state that the study is a prospective observational study.*

**Response:** We have made changes in title to match the Methods of our study:

“Transforaminal Pulsed radiofrequency and Epidural steroid injection on chronic lumbar radiculopathy: A prospective observational study from a tertiary care hospital in Vietnam.” (Title page)

*3. Reference should be added for the "Study procedure". Has this procedure been applied in accordance with the literature?*

**Response:** We have added more details:

“The stepwise procedure was conducted, according to the practice guidelines of the International Spine Intervention Society[6]” (page: 5, line: 92-93)

6. International Spinal Intervention Society. Lumbar transforaminal injections. In: Bogduk N, ed. Practice Guidelines for Spinal Diagnostic and Treatment Procedures. San Francisco, CA: International Spinal Inter-vention Society; 2004:163–87.

*4. Spondylolisthesis is included in the exclusion criteria, but in the result section, it is written that 12 (15.8%) patients have Spondylolisthesis. This is a contradiction.*

**Response:** We have added missing information:

“Spinal tumors, tuberculosis, spondylolisthesis (grade ≥ II), and fracture spine.” (page 4, line: 71)

**Thank you very much for your comments and suggestions!**
