# Supplementary material for: Transforaminal pulsed radiofrequency and epidural steroid injection on chronic lumbar radiculopathy: A prospective observational study from a tertiary care hospital in Vietnam
Source: PLoS One. 2024 Apr 4;19(4):e0292042. doi: 10.1371/journal.pone.0292042 (PMC10994338; doi:10.1371/journal.pone.0292042)
Supplement: Attachment — Submitted filename: reviewer comment plosone.docx [file pone.0292042.s005.docx]

It is written as "neuropathic pain" in the abstract (conclusion) part. However, there is no neuropathic pain assessment scale in the material method section. For this reason, it would be more appropriate to say "pain" instead of neuropathic pain.

The phrase "A case series" in the title partially contradicts the statement "A prospective observational study" in the material method section. The title or abstract should state that the study is a prospective observational study.

Reference should be added for the "Study procedure". Has this procedure been applied in accordance with the literature?

Spondylolisthesis is included in the exclusion criteria, but in the result section, it is written that 12 (15.8%) patients have Spondylolisthesis. This is a contradiction.

Thanks
